# Systematic Differences in Impact across Publication Tracks at PNAS

**DOI:** 10.1371/journal.pone.0008092

**Published:** 2009-12-01

**Authors:** David G. Rand, Thomas Pfeiffer

**Affiliations:** 1 Program for Evolutionary Dynamics, Harvard University, Cambridge, Massachusetts, United States of America; 2 Berkman Center for Internet and Society, Harvard University, Cambridge, Massachusetts, United States of America; University of Exeter, United Kingdom

## Abstract

**Background:**

Citation data can be used to evaluate the editorial policies and procedures of scientific journals. Here we investigate citation counts for the three different publication tracks of the Proceedings of the National Academy of Sciences of the United States of America (PNAS). This analysis explores the consequences of differences in editor and referee selection, while controlling for the prestige of the journal in which the papers appear.

**Methodology/Principal Findings:**

We find that papers authored and “Contributed” by NAS members (Track III) are on average cited less often than papers that are “Communicated” for others by NAS members (Track I) or submitted directly via the standard peer review process (Track II). However, we also find that the variance in the citation count of Contributed papers, and to a lesser extent Communicated papers, is larger than for direct submissions. Therefore when examining the 10% most-cited papers from each track, Contributed papers receive the most citations, followed by Communicated papers, while Direct submissions receive the least citations.

**Conclusion/Significance:**

Our findings suggest that PNAS “Contributed” papers, in which NAS–member authors select their own reviewers, balance an overall lower impact with an increased probability of publishing exceptional papers. This analysis demonstrates that different editorial procedures are associated with different levels of impact, even within the same prominent journal, and raises interesting questions about the most appropriate metrics for judging an editorial policy's success.

## Introduction

Citation data play an important role in the evaluation of scientific research. Citation counts can be used to characterize individual studies and researchers, scientific disciplines, journals, institutions, and entire nations [1]–[5]. At the level of scientific journals, citation data can help to investigate the effect of review policies and procedures on the subsequent impact of published papers [6], [7]. The Proceedings of the National Academy of Sciences (PNAS) offers a particularly attractive subject of study for peer review procedures, due to its three different publication tracks, each with its own set of procedures.

The majority of papers published in PNAS are submitted directly to the journal and follow the standard peer review process. The editorial board appoints an editor for each Direct submission, who then solicits reviewers. During the review process the authors are blinded to the identities of both the editor and the referees. PNAS refers to this publication method as “Track II”. In addition to the direct submission track, members of the National Academy of Sciences (NAS) are allowed to “Communicate” up to two papers per year for other authors. Here, authors send their paper to the NAS member, who then procures reviews from at least two other researchers and submits the paper and reviews to the PNAS editorial board for approval. As with Direct submissions, authors of Communicated papers are at least in theory blinded to the identity of their reviewers, but not to the identity of the editor. PNAS refers to this publication method as “Track I”. Lastly, NAS members are allowed to “Contribute” as many of their own papers per year as they wish. Here, NAS members choose their own referees, collect at least two reviews, and submit their paper along with the reviews to the PNAS editorial board. Peer review is no longer blind, as the authoring NAS member selects his or her own reviewers. PNAS refers to this publication method as “Track III”. For more information on the different PNAS publication tracks, see [8]. Examining papers published in PNAS provides an opportunity to evaluate how these differences in the submission and peer review process within the same journal affect the impact of the papers finally published. The possibility that impact varies systematically across track has received a great deal of recent attention, particularly in light of the decision by PNAS to discontinue Track I [9]. The citation analysis we now present provides a quantitative treatment of the quality of papers published through each track, a discussion which as hitherto been largely anecdotal in nature.

## Methods

To empirically investigate the impact of papers published via each track, we inspect 2695 papers published between June 1, 2004 and April 26, 2005, covering PNAS Volume 101 Issue 22 through Volume 102 Issue 17. For each paper, we examine Thomson Reuters Web of Science citation data as of October 2006 and May 2009, as well as page-view counts as of October 2006. We also note the track through which each paper was published, the topic classification of each paper, the date of publication, and whether each article was published as open access and/or as part of a special feature. For all subsequent analysis, we log10-transform citation counts, with the addition of a constant value 1 to all citation entries so as not to exclude un-cited papers. Because citation rates come from a long-tailed distribution with the most cited papers attracting many more citations than the average paper, this transformation brings the citation count distributions much closer to normal (see Text S1 for summary statistics and distribution histograms).

## Results

**Figure 1:**
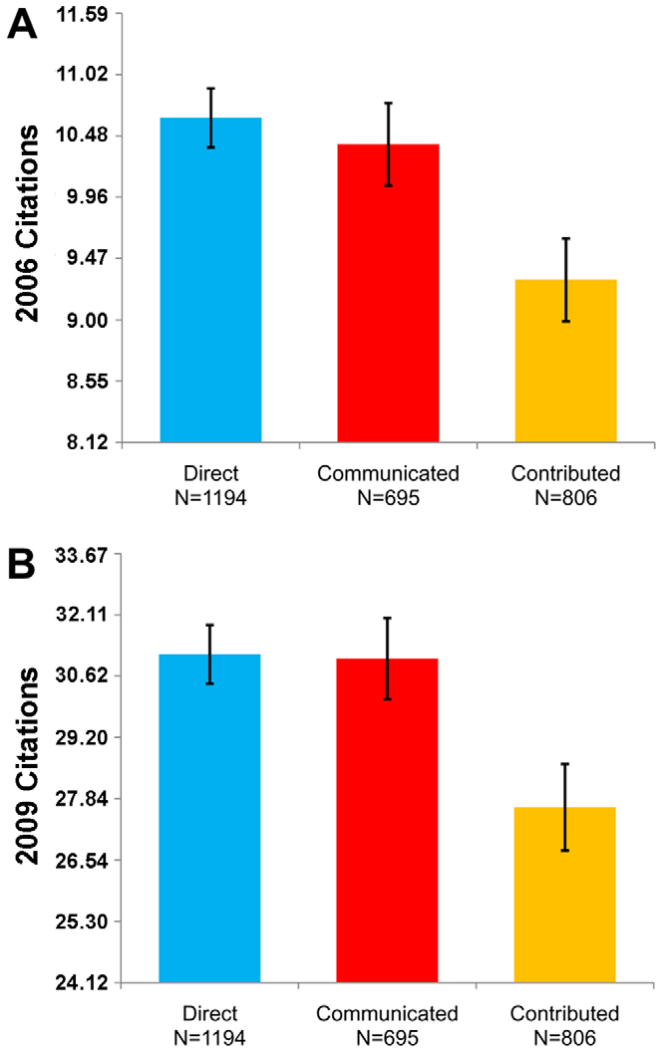
Citation counts by track. Data from October 2006 (A) and May 2009 (B). Contributed papers are cited significantly less on average than Direct submissions or Communicated papers. Citation count distributions for each Track are shown in Text S1. The median 2006 citation count is 11 for Direct submissions, 11 for Communicated papers, and 9.5 for Contributed papers. The median 2009 citation count is 32.5 for Direct submissions, 31 for Communicated papers, and 28 for Contributed papers. Error bars indicate standard error of the mean. Note that the mean and the standard error of the mean are calculated from log10-transformed citation counts.

As a first, most straightforward analysis, we compare average citation counts across tracks (Figure 1). Using both 2006 and 2009 citation counts, we find no significant difference between Direct submissions and Communicated papers (Ranksum, 2006: p = 0.68, 2009: p = 0.78), while Contributed papers are cited significantly less on average than either other track (Ranksum, Direct vs Contributed: 2006, p = 0.0007; 2009, p = 0.002; Communicated vs Contributed: 2006, p = 0.011; 2009, p = 0.013). The median 2006 citation count is 11 for Direct submissions, 11 for Communicated papers, and 9.5 for Contributed papers. The median 2009 citation count is 32.5 for Direct submissions, 31 for Communicated papers, and 28 for Contributed papers.

Comparing the variance in citation counts across Tracks, however, reveals that there is significantly greater variation in the impact of Contributed papers compared to Direct submissions or Communicated papers (Levene's F-Test for homogeneity of variances, Direct vs Contributed: 2006, p = 0.0001; 2009, p<0.0001; Communicated vs Contributed: 2006, p = 0.10; 2009, p = 0.002), and marginally greater variation in Communicated papers relative to Direct submissions (Levene's F-Test for homogeneity of variances, 2006, p = 0.058; 2009, p = 0.15). To explore the consequences of this larger variance, we now compare the citation counts of the 10% least and most cited papers from each Track.

**Figure 2:**
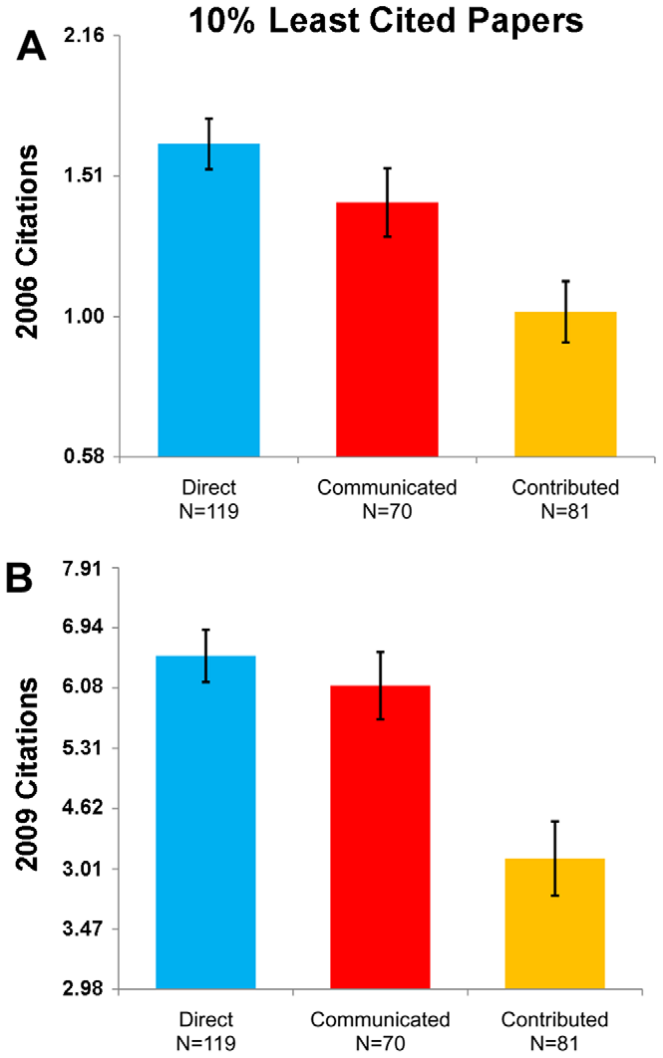
Citation counts of the 10% least cited papers in each track. Data from October 2006 (A) and May 2009 (B). The least cited Contributed papers are cited significantly less on average than the least cited Direct submissions or Communicated papers. The range of 2006 citations counts for the 10% least cited Direct submissions is 0–8, Communicated papers 0–7, and Contributed papers 0–5. The range of 2009 citation counts for the 10% least cited Direct submissions is 0–11, Communicated papers 0–11, and Contributed papers 0–9. Error bars indicate standard error of the mean. Note that the mean and the standard error of the mean are calculated from log10-transformed citation counts.

Among the 10% least cited papers in each track, we see similar results to the analysis comparing citation counts among all papers (Figure 2). There is no significant difference in citations between the bottom 10% of Communicated papers and the bottom 10% of Direct submissions (Ranksum, 2006, p = 0.105; 2009, p = 0.33), while the bottom 10% of Contributed papers receive significantly fewer citations than the bottom 10% of Direct submissions (Ranksum, 2006, p<0.0001; 2009, p<0.0001). To help illustrate this point, the range of 2006 citations counts for the 10% least cited Direct submissions is 0–8, Communicated papers 0–7, and Contributed papers 0–5; the range of 2009 citation counts for the 10% least cited Direct submissions is 0–11, Communicated papers 0–11, and Contributed papers 0–9.

**Figure 3:**
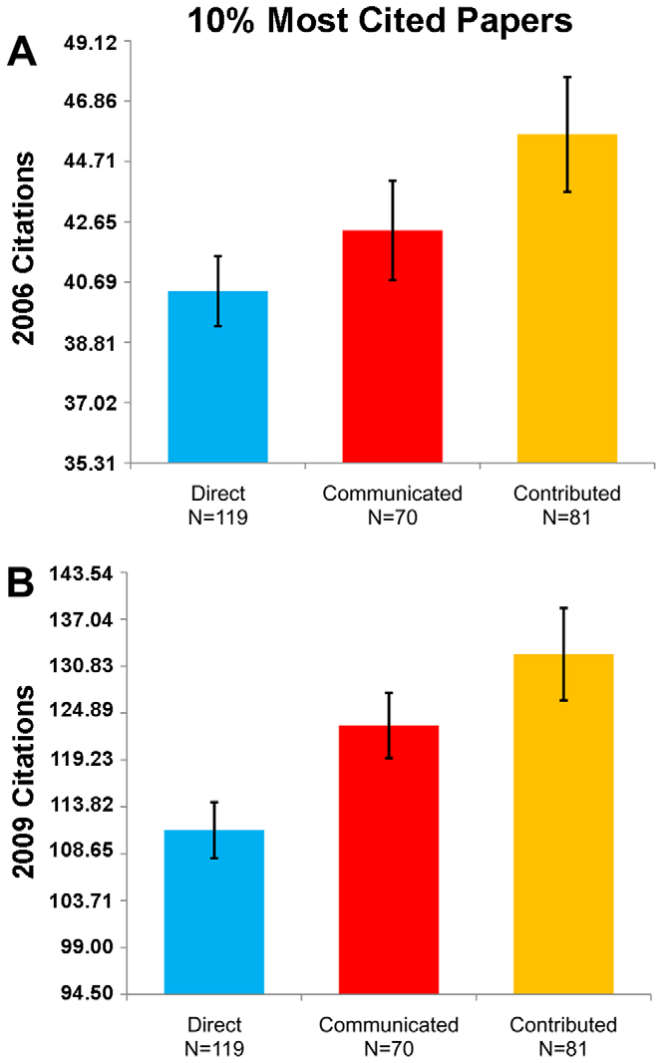
Citation counts of the 10% most cited papers in each track. Data from October 2006 (A) and May 2009 (B). The top Contributed papers are cited the most, followed by the top Communicated papers, and the top Direct submissions are cited least. The range of 2006 citations counts for the 10% most cited Direct submissions is 28–102, Communicated papers 30–129, and Contributed papers 30–422. The range of 2009 citation counts for the 10% most cited Direct submissions is 81–403, Communicated papers 87–313, and Contributed papers 87–975. Error bars indicate standard error of the mean. Note that the mean and the standard error of the mean are calculated from log10-transformed citation counts.

Among the 10% most cited papers in each track, however, we find the opposite pattern (Figure 3). The top 10% of Contributed papers receive significantly more citations than the top 10% of Direct submissions (Ranksum, 2006, p = 0.022; 2009, p = 0.0001). The top 10% of Communicated papers also receive more citations than the top 10% of Direct submissions, although the difference is only significant in the 2009 citation data (Ranksum, 2006, p = 0.33; 2009, p = 0.001). To help illustrate this point, the range of 2006 citations counts for the 10% most cited Direct submissions is 28–102, Communicated papers 30–129, and Contributed papers 30–422; the range of 2009 citation counts for the 10% most cited Direct submissions is 81–403, Communicated papers 87–313, and Contributed papers 87–975.

We now demonstrate that the relationships shown in Figure 1, Figure 2, and Figure 3 are robust to controlling for a number of additional factors which may affect citation counts. Open access publication [10]–[12] has been suggested to affect impact, as has time since publication and topic classification [13]. Therefore we control for these factors, as well as publication as part of a special feature issue. We use regression to a linear model with robust standard errors [14] using log-transformed citation rate as the dependent variable, and submission track as well as the above mentioned factors as independent variables. An overview of the variables and the levels each can take is given in Text S1.

Our additional analyses reproduce the effects demonstrated by our comparison of means above. For full regression tables, see Text S1. Considering all 2695 papers, there is no significant difference in 2006 or 2009 citation counts between Communicated papers and Direct submissions (2006: coeff = −0.010, p = 0.532; 2009: coeff = −0.014, p = 0.398), while Contributed papers are cited significantly less than Direct submissions (2006: coeff = −0.050, p = 0.002; 2009: coeff = −0.056, p = 0.001). Given the logarithmic scaling of citation counts, these regression coefficients indicate that Contributed papers receive approximately 10% fewer citations than Direct submissions. It is also interesting to note that, similar to previous observations [10]–[12], Open Access papers receive approximately 25% more citations than non-Open Access papers (Median 2006 [2009] citations: Open access = 12.5 [38], non-Open access = 10 [30]; 75% percentile 2006 [2009] citations: Open access = 21 [61], non-Open access = 17 [50]).

Similarly, considering only the 10% least cited papers from each track, there is no significant difference between Communicated papers and Direct submissions (2006: coeff = −0.019, p = 0.558; 2009: coeff = −0.014, p = 0.725), while Contributed papers are cited significantly less than Direct submissions (2006: coeff = −0.087, p = 0.004; 2009: coeff = −0.147, p<0.001). These regression coefficients indicate that when only considering the least cited papers, Contributed papers receive approximately 20% fewer citations than Direct submissions in the 2006 citation count, and approximately 30% fewer citations in the 2009 citation count.

Considering only the 10% most cited papers from each track, however, Communicated papers are cited significantly more than Direct submissions in 2009 but not 2006 (2006: coeff = 0.029, p = 0.176; 2009: coeff = 0.050, p = 0.012), and Contributed papers are cited significantly more than Direct submissions in both 2006 and 2009 (2006: coeff = 0.059, p = 0.006; 2009: coeff = 0.073, p = 0.001). These regression coefficients indicate that Contributed papers receive approximately 10% more citations than Direct submissions when considering only the most cited papers.

In addition to citation counts, the number of page-views received by each article provide another measure of impact. However, examining page-view counts as of October 2006 shows no significant variation across Tracks when controlling for open access publication, inclusion in a special feature, days since publication and topic classification. More current page-view statistics may shed greater light on differences between Tracks if differences in page-views are amplified over time. This data, however, was not available to us. Among the 10% most viewed papers in each track, page-views follow the same pattern as citation counts, with Communicated and Contributed papers receiving significantly more page-views than Direct submissions. See Text S1 for details.

## Discussion

The analysis presented here clearly demonstrates variation in impact among papers published using different review processes at PNAS. We find that overall, papers authored by NAS member and Contributed to PNAS are cited significantly less than papers which are Direct submissions. Strikingly, however, we find that the 10% most cited Contributed papers receive significantly *more* citations than the 10% most cited Direct submissions. Thus the Contributed track seems to yield less influential papers on average, but is more likely produce truly exceptional papers. In addition, we find no significant difference in overall citation count between papers submitted directly to PNAS and papers Communicated for others by NAS members, while the 10% most cited Communicated papers also receive significantly more citations than the 10% most cited Direct submissions.

What might be responsible for these differences in impact between the different publication tracks at PNAS? It is possible for NAS members to Communicate or Contribute papers in fields outside their area of expertise, and to soften the challenges of the peer review process through their choice of referees. The anonymity of referees and reviewers is also decreased for Communicated and Contributed papers relative to Direct submissions. These factors could result in lower quality papers being submitted and published through these alternative publication avenues. Our analysis suggests that this may in fact happen for Contributed papers, but does not support the suggestion that papers Communicated by NAS members are on average of lower quality than Direct submissions. While it is still possible that more low-quality papers are submitted through the Communication track, our results suggest that the PNAS Editorial Board successfully screens out such papers.

In contrast to these potential dangers associated with Communicated and Contributed papers, it is also possible that these alternative publishing procedures may facilitate the publication of time-sensitive and groundbreaking work which is of high quality but might suffer under the standard review process. Our analysis supports this hypothesis, by showing that the most cited Contributed and Communicated papers are more influential than the top Direct submissions. The benefit of facilitating publication of extremely high-impact Contributed papers could be argued to out-weigh the potential cost of allowing more low quality papers to also be published.

Together, these two observations about Contributed papers raise interesting questions about the optimal publication procedures, as well as the appropriate metric for assessing optimality. Editors at some journals may seek to maximize the overall impact of the papers they publish, while others may be willing to accept lower median impact in exchange for increased impact among the most influential papers. Our results suggest that making publication easier for researchers with established track records may have the latter effect. Which strategy is most appropriate or effective remains an open question, and one which may not have a normative answer. Further empirical and theoretical work exploring these questions is needed.

One additional factor which may influence citation counts is whether a given paper was highlighted in a PNAS press release. We were unable to explore this issue because of lack of data regarding which papers received press releases. The effect of press releases, and popular press coverage more generally, on citation counts is an open question which deserves further study [2]. A related issue involves the potential for selection bias in which papers are submitted to PNAS via each track. In addition to the differences in referee selection and review process, authors may choose to submit papers they feel are stronger or weaker through particular tracks. The direction of this effect, however, is unclear. For example, one could hypothesize that weaker papers are submitted through alternative tracks to increase the probability of acceptance, or that stronger papers are submitted through alternative tracks to increase the speed of acceptance. Or perhaps both suggestions are correct. Exploring this issue also merits future study.

The empirical analysis presented here clearly demonstrates systematic variation in citation counts across publication tracks at PNAS. Differences in the submission and review process between Direct submissions, Communicated papers and Contributed papers seem to have significant effects on the influence of papers published in PNAS. As well as being of interest to decision makers at, and readers of, PNAS, these results have potentially important implications for those making decisions about submission and review procedures at other journals, and raise interesting questions about how editors should evaluate a journal's success. Quantitative comparisons of impact across similar journals with different publication processes represent an important opportunity for exploring publication dynamics which lie at the heart of the modern scientific enterprise.

## Supporting Information

Text S1 Supplementary file containing additional analysis and figures.
(0.47 MB DOC)
