# Supplementary material for: Systematic Differences in Impact across Publication Tracks at PNAS
Source: PLoS One. 2009 Dec 1;4(12):e8092. doi: 10.1371/journal.pone.0008092 (PMC2778996; doi:10.1371/journal.pone.0008092)
Supplement: Text S1 — Supplementary file containing additional analysis and figures. (0.47 MB DOC) [file pone.0008092.s001.doc]

**Systematic differences in impact across publication tracks at PNAS**

**Supporting Information S1**

David G. Rand^1,2*^ & Thomas Pfeiffer^1^

^1^Program for Evolutionary Dynamics and ^2^Berkman Center for Internet and Society, Harvard University, Cambridge MA 02138

**Contents**

1. Summary statistics

2. Distribution of citation counts by Track

3. Analysis of citation counts controlling for additional factors

4. Analysis of page-views as of October 2006

1. Summary Statistics

| **Table S1.** Summary statistics. N=2695 papers. | | | | | |
| --- | --- | --- | --- | --- | --- |
| Variable | Levels | Mean | Std. Dev. | Min | Max |
| log10(Citations2006+1) |  | 1.048 | 0.355 | 0 | 2.626 |
| log10(Citations2009+1) |  | 1.492 | 0.358 | 0 | 2.989 |
| log10(Pageviews2006) |  | 3.403 | 0.230 | 2.658 | 4.669 |
| Age (Days relative to newest article) |  | 162 | 98 | 0 | 329 |
| Submission track | Direct submission | 44.3% |  |  |  |
|  | Communicated | 25.8% |  |  |  |
|  | Contributed | 29.9% |  |  |  |
| Topic classification | Agricultural Sciences | 0.26% |  |  |  |
|  | Anthropology | 0.41% |  |  |  |
|  | Applied Biological Sciences | 1.52% |  |  |  |
|  | Biochemistry | 12.62% |  |  |  |
|  | Biophysics and Computational Biology | 8.57% |  |  |  |
|  | Cell Biology | 7.42% |  |  |  |
|  | Developmental Biology | 3.23% |  |  |  |
|  | Ecology | 2.08% |  |  |  |
|  | Environmental Sciences | 0.15% |  |  |  |
|  | Evolution | 5.05% |  |  |  |
|  | Genetics | 6.20% |  |  |  |
|  | Immunology | 5.97% |  |  |  |
|  | Medical Sciences | 10.13% |  |  |  |
|  | Microbiology | 6.60% |  |  |  |
|  | Neuroscience | 11.17% |  |  |  |
|  | Pharmacology | 1.60% |  |  |  |
|  | Physiology | 2.63% |  |  |  |
|  | Plant Biology | 3.27% |  |  |  |
|  | Population Biology | 0.41% |  |  |  |
|  | Psychology | 0.82% |  |  |  |
|  | Physical Science | 8.8% |  |  |  |
|  | Social Science | 1.1% |  |  |  |
| Open Access | Yes | 14.1% |  |  |  |
|  | No | 85.9% |  |  |  |
| Special Feature | Yes | 1.3% |  |  |  |
|  | No | 98.7% |  |  |  |

2. Distribution of citation counts by Track

| **Table S2.** 2006 Citation counts | | | | | | |
| --- | --- | --- | --- | --- | --- | --- |
|  |  | Direct |  | Communicated |  | Contributed |
| 10% |  | 3 |  | 3 |  | 2 |
| 25% |  | 6 |  | 6 |  | 5 |
| 50% |  | 11 |  | 11 |  | 9.5 |
| 75% |  | 18 |  | 18 |  | 17 |
| 90% |  | 28 |  | 30 |  | 30 |

| **Table S3.** 2009 Citations counts | | | | | | |
| --- | --- | --- | --- | --- | --- | --- |
|  |  | Direct |  | Communicated |  | Contributed |
| 10% |  | 11 |  | 11 |  | 9 |
| 25% |  | 20 |  | 19 |  | 15 |
| 50% |  | 32.5 |  | 31 |  | 28 |
| 75% |  | 51 |  | 53 |  | 51 |
| 90% |  | 80 |  | 87 |  | 87 |

**
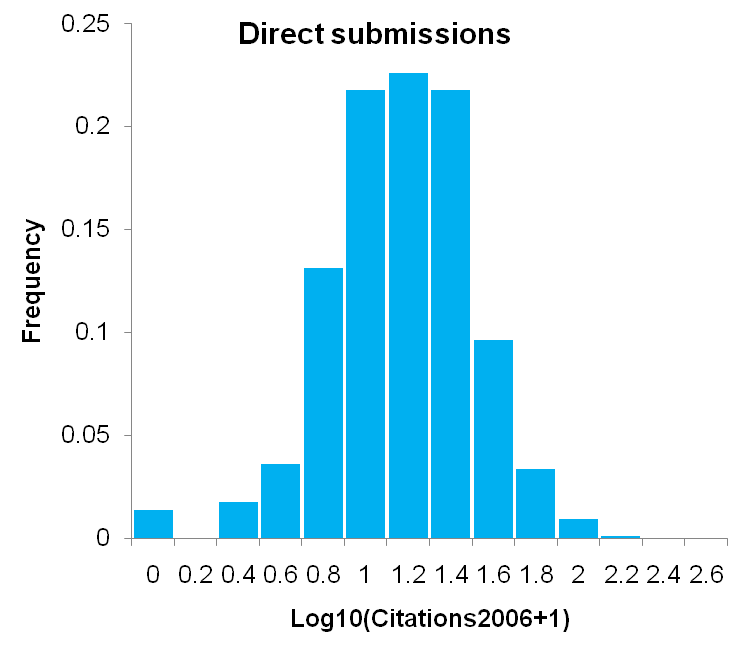

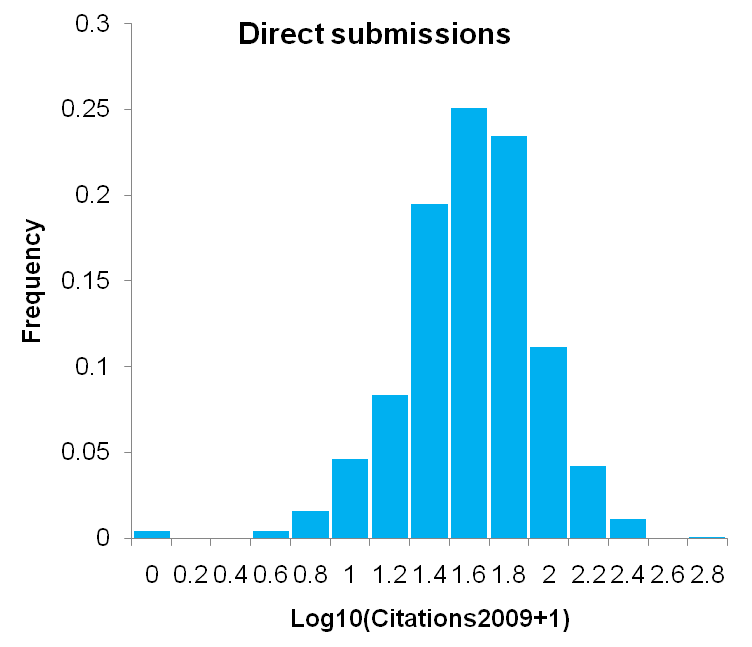
**

**
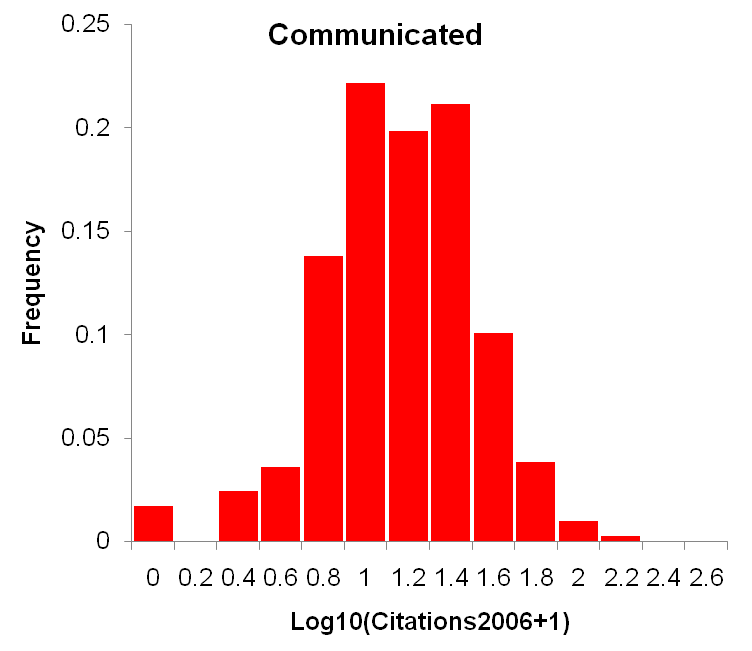

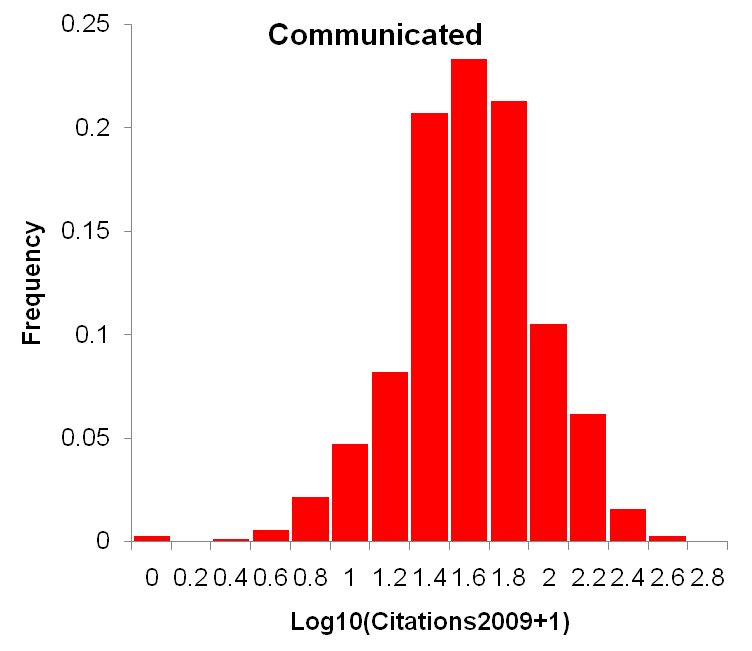
**

**
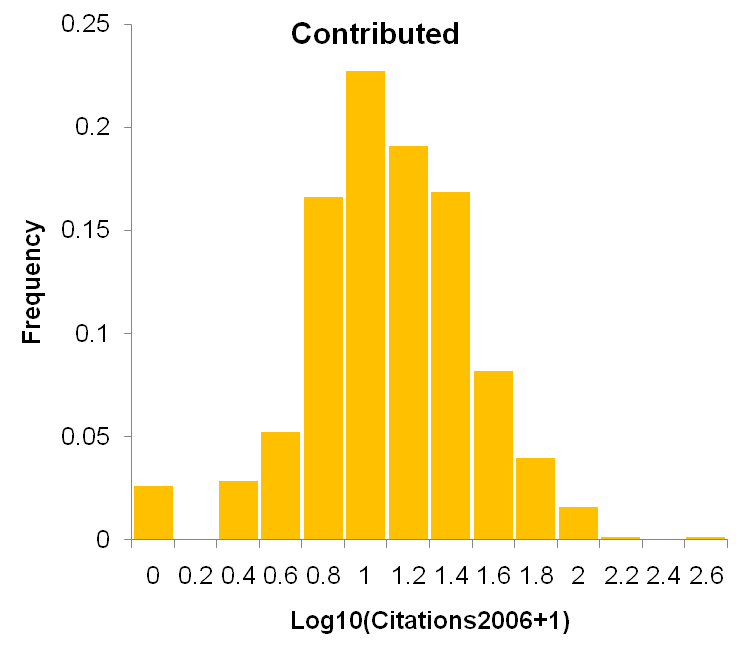

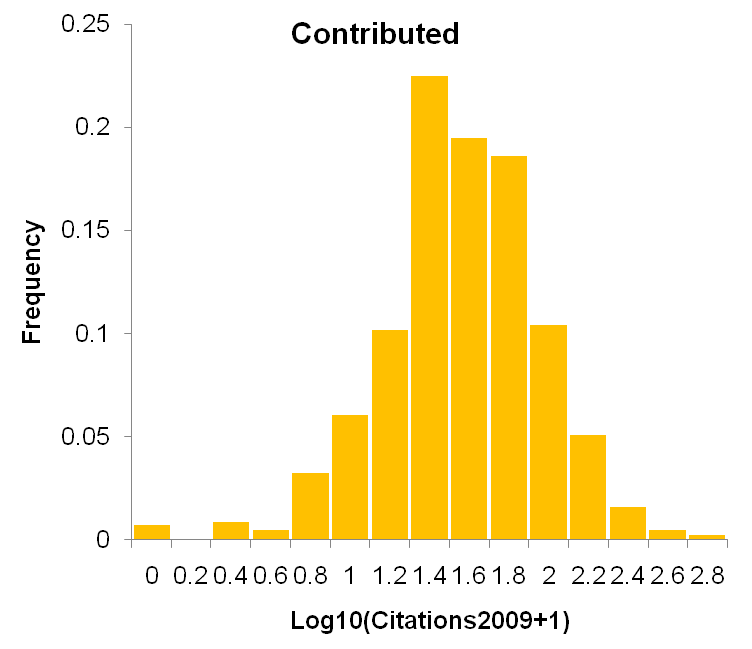
**

**Figure S1.** Distributions of Log10(Citation06+1) (First column) and Log10(Citations2009+1) (Second Column) by Track.

*3. Analysis of citation counts controlling for additional factors*

Here we use regression to a standard linear model, with log-transformed citation counts as the dependent variable, and submission track, open access, special feature and field as independent variables. Coefficients for Communicated and Contributed levels of submission track are relative to Direct submissions. Coefficients for each subfield are relative to biochemistry (the most common subfield).

| **Table S4.** Regression on 2006 Citations. | | | | |
| --- | --- | --- | --- | --- |
|  | Coefficient | Test statistic | | P-value |
| Communicated | -0.010 | -0.63 | | 0.532 |
| Contributed | -0.050 | -3.17 | | 0.002 |
| Open Access | 0.104 | 5.43 | | <0.001 |
| Special Feature | -0.022 | -0.39 | | 0.694 |
| Age (Days relative to newest article) | 0.0008 | 12.43 | | <0.001 |
| Agricultural Sciences | 0.082 | 0.58 | | 0.563 |
| Anthropology | -0.184 | -2.71 | | 0.007 |
| Applied Biological Sciences | 0.040 | 0.69 | | 0.491 |
| Biophysics and Computational Biology | 0.069 | 2.71 | | 0.007 |
| Cell Biology | 0.089 | 3.04 | | 0.002 |
| Developmental Biology | 0.021 | 0.58 | | 0.56 |
| Ecology | -0.057 | -1.05 | | 0.293 |
| Environmental Sciences | 0.020 | 0.12 | | 0.904 |
| Evolution | 0.031 | 0.91 | | 0.365 |
| Genetics | 0.113 | 3.75 | | <0.001 |
| Immunology | 0.081 | 2.47 | | 0.014 |
| Medical Sciences | 0.133 | 5.23 | | <0.001 |
| Microbiology | 0.149 | 4.92 | | <0.001 |
| Neuroscience | 0.033 | 1.32 | | 0.187 |
| Pharmacology | 0.055 | 1.03 | | 0.302 |
| Physiology | -0.018 | -0.42 | | 0.677 |
| Plant Biology | 0.101 | 2.88 | | 0.004 |
| Population Biology | -0.139 | -1.40 | | 0.162 |
| Psychology | -0.139 | -1.79 | | 0.074 |
| Physical Science | -0.148 | -4.61 | | <0.001 |
| Social Science | -0.364 | -5.61 | | <0.001 |
| Intercept | 0.880 | 40.24 | | <0.001 |
| N=2695, R^2^=0.127. Ordinary least squares regression with robust standard errors. For binary variables, 0=No 1=Yes. | | | | |
| Dependent variable: Log10(Citations2006+1) | |  |  | |

| **Table S5.** Regression on 2009 Citations. | | | | |
| --- | --- | --- | --- | --- |
|  | Coefficient | Test statistic | | P-value |
| Communicated | -0.014 | -0.85 | | 0.398 |
| Contributed | -0.056 | -3.37 | | 0.001 |
| Open Access | 0.111 | 5.47 | | <0.001 |
| Special Feature | -0.084 | -1.58 | | 0.114 |
| Age (Days relative to newest article) | 0.0003 | 4.22 | | <0.001 |
| Agricultural Sciences | 0.249 | 1.75 | | 0.081 |
| Anthropology | -0.057 | -1.31 | | 0.189 |
| Applied Biological Sciences | 0.127 | 1.70 | | 0.09 |
| Biophysics and Computational Biology | 0.097 | 3.49 | | <0.001 |
| Cell Biology | 0.126 | 4.19 | | <0.001 |
| Developmental Biology | 0.056 | 1.51 | | 0.132 |
| Ecology | 0.118 | 2.43 | | 0.015 |
| Environmental Sciences | 0.196 | 0.99 | | 0.321 |
| Evolution | 0.074 | 2.17 | | 0.03 |
| Genetics | 0.129 | 3.94 | | <0.001 |
| Immunology | 0.111 | 3.42 | | 0.001 |
| Medical Sciences | 0.178 | 6.43 | | <0.001 |
| Microbiology | 0.149 | 4.67 | | <0.001 |
| Neuroscience | 0.081 | 3.06 | | 0.002 |
| Pharmacology | 0.126 | 2.59 | | 0.01 |
| Physiology | 0.032 | 0.67 | | 0.504 |
| Plant Biology | 0.150 | 4.07 | | <0.001 |
| Population Biology | -0.021 | -0.22 | | 0.823 |
| Psychology | -0.040 | -0.50 | | 0.619 |
| Physical Science | -0.079 | -2.30 | | 0.022 |
| Social Science | -0.252 | -3.25 | | 0.001 |
| Intercept | 1.373 | 57.46 | | <0.001 |
| N=2695, R^2^=0.074. Ordinary least squares regression with robust standard errors. For binary variables, 0=No 1=Yes. | | | | |
| Dependent variable: Log10(Citations2009+1) | |  |  | |

| **Table S6.** Regression on 10% least cited papers from each track, 2006 Citations. | | | | |
| --- | --- | --- | --- | --- |
|  | Coefficient | Test statistic | | P-value |
| Communicated | -0.019 | -0.59 | | 0.558 |
| Contributed | -0.087 | -2.88 | | 0.004 |
| Open Access | -0.034 | -0.82 | | 0.415 |
| Special Feature | 0.082 | 1.07 | | 0.287 |
| Age (Days relative to newest article) | 0.0001 | 0.46 | | 0.649 |
| Agricultural Sciences | 0.026 | 0.62 | | 0.535 |
| Anthropology | 0.005 | 0.10 | | 0.916 |
| Applied Biological Sciences | -0.134 | -1.40 | | 0.162 |
| Biophysics and Computational Biology | 0.039 | 0.81 | | 0.42 |
| Cell Biology | -0.034 | -0.53 | | 0.595 |
| Developmental Biology | -0.006 | -0.10 | | 0.921 |
| Ecology | -0.094 | -1.27 | | 0.204 |
| Environmental Sciences | - |  | |  |
| Evolution | 0.027 | 0.51 | | 0.612 |
| Genetics | 0.058 | 1.28 | | 0.201 |
| Immunology | -0.097 | -1.35 | | 0.178 |
| Medical Sciences | -0.008 | -0.13 | | 0.893 |
| Microbiology | -0.015 | -0.28 | | 0.784 |
| Neuroscience | -0.041 | -0.78 | | 0.438 |
| Pharmacology | -0.115 | -2.59 | | 0.01 |
| Physiology | -0.218 | -1.85 | | 0.065 |
| Plant Biology | -0.007 | -0.06 | | 0.949 |
| Population Biology | 0.131 | 3.00 | | 0.003 |
| Psychology | -0.146 | -3.78 | | <0.001 |
| Physical Science | -0.162 | -3.45 | | 0.001 |
| Social Science | -0.137 | -1.92 | | 0.056 |
| Intercept | 0.453 | 10.02 | | <0.001 |
| N=270, R^2^=0.194. Ordinary least squares regression with robust standard errors. For binary variables, 0=No 1=Yes. Variables for topics which have no papers in the 2006 top 10% are dropped. | | | | |
| Dependent variable: Log10(Citations2006+1) | |  |  | |

| **Table S7.** Regression on 10% least cited papers from each track, 2009 Citations. | | | | |
| --- | --- | --- | --- | --- |
|  | Coefficient | Test statistic | | P-value |
| Communicated | -0.014 | -0.35 | | 0.725 |
| Contributed | -0.147 | -3.70 | | <0.001 |
| Open Access | 0.078 | 1.60 | | 0.11 |
| Special Feature | 0.173 | 2.98 | | 0.003 |
| Age (Days relative to newest article) | 0.0000 | -0.13 | | 0.9 |
| Agricultural Sciences | 0.028 | 0.68 | | 0.497 |
| Anthropology | - |  | |  |
| Applied Biological Sciences | -0.084 | -0.58 | | 0.563 |
| Biophysics and Computational Biology | 0.034 | 0.76 | | 0.451 |
| Cell Biology | -0.071 | -0.76 | | 0.448 |
| Developmental Biology | 0.055 | 1.16 | | 0.245 |
| Ecology | 0.014 | 0.19 | | 0.85 |
| Environmental Sciences | - |  | |  |
| Evolution | 0.031 | 0.65 | | 0.515 |
| Genetics | 0.044 | 0.73 | | 0.469 |
| Immunology | -0.046 | -0.49 | | 0.626 |
| Medical Sciences | -0.144 | -1.18 | | 0.241 |
| Microbiology | -0.013 | -0.15 | | 0.878 |
| Neuroscience | -0.011 | -0.21 | | 0.838 |
| Pharmacology | 0.169 | 3.48 | | 0.001 |
| Physiology | -0.169 | -1.39 | | 0.166 |
| Plant Biology | -0.253 | -0.91 | | 0.363 |
| Population Biology | 0.191 | 4.89 | | <0.001 |
| Psychology | 0.081 | 1.04 | | 0.299 |
| Physical Science | -0.101 | -1.92 | | 0.056 |
| Social Science | -0.160 | -1.73 | | 0.085 |
| Intercept | 0.894 | 15.79 | | <0.001 |
| N=270, R^2^=0.174. Ordinary least squares regression with robust standard errors. For binary variables, 0=No 1=Yes. Variables for topics which have no papers in the 2009 top 10% are dropped. | | | | |
| Dependent variable: Log10(Citations2009+1) | |  |  | |

| **Table S8.** Regression on 10% most cited papers from each track, 2006 Citations. | | | | |
| --- | --- | --- | --- | --- |
|  | Coefficient | Test statistic | | P-value |
| Communicated | 0.029 | 1.36 | | 0.176 |
| Contributed | 0.059 | 2.76 | | 0.006 |
| Open Access | 0.025 | 0.80 | | 0.426 |
| Special Feature | - |  | |  |
| Age (Days relative to newest article) | 0.0003 | 2.70 | | 0.007 |
| Agricultural Sciences | 0.020 | 0.53 | | 0.597 |
| Anthropology | - |  | |  |
| Applied Biological Sciences | 0.114 | 0.57 | | 0.568 |
| Biophysics and Computational Biology | -0.005 | -0.15 | | 0.88 |
| Cell Biology | 0.015 | 0.43 | | 0.669 |
| Developmental Biology | 0.068 | 1.12 | | 0.263 |
| Ecology | 0.055 | 0.77 | | 0.44 |
| Environmental Sciences | - |  | |  |
| Evolution | 0.036 | 0.97 | | 0.334 |
| Genetics | 0.018 | 0.54 | | 0.593 |
| Immunology | 0.055 | 1.48 | | 0.141 |
| Medical Sciences | 0.038 | 0.87 | | 0.387 |
| Microbiology | 0.102 | 2.57 | | 0.011 |
| Neuroscience | -0.007 | -0.20 | | 0.844 |
| Pharmacology | 0.041 | 0.48 | | 0.631 |
| Physiology | -0.042 | -1.34 | | 0.181 |
| Plant Biology | 0.038 | 0.71 | | 0.481 |
| Population Biology | -0.067 | -1.68 | | 0.095 |
| Psychology | 0.183 | 4.52 | | <0.001 |
| Physical Science | 0.020 | 0.39 | | 0.695 |
| Social Science | - |  | |  |
| Intercept | 1.522 | 42.20 | | <0.001 |
| N=270, R^2^=0.115. Ordinary least squares regression with robust standard errors. For binary variables, 0=No 1=Yes. Variables for topics which have no papers in the 2006 top 10% are dropped. | | | | |
| Dependent variable: Log10(Citations2006+1) | |  |  | |

| **Table S9.** Regression on 10% most cited papers from each track, 2009 Citations. | | | | |
| --- | --- | --- | --- | --- |
|  | Coefficient | Test statistic | | P-value |
| Communicated | 0.050 | 2.52 | | 0.012 |
| Contributed | 0.073 | 3.26 | | 0.001 |
| Open Access | 0.052 | 1.76 | | 0.08 |
| Special Feature | - |  | |  |
| Age (Days relative to newest article) | 0.0001 | 1.34 | | 0.183 |
| Agricultural Sciences | -0.081 | -2.09 | | 0.037 |
| Anthropology | - |  | |  |
| Applied Biological Sciences | -0.027 | -0.32 | | 0.746 |
| Biophysics and Computational Biology | -0.037 | -1.06 | | 0.289 |
| Cell Biology | -0.034 | -0.98 | | 0.329 |
| Developmental Biology | 0.078 | 1.19 | | 0.235 |
| Ecology | 0.019 | 0.29 | | 0.771 |
| Environmental Sciences | -0.005 | -0.15 | | 0.882 |
| Evolution | 0.017 | 0.41 | | 0.681 |
| Genetics | 0.022 | 0.53 | | 0.598 |
| Immunology | 0.026 | 0.74 | | 0.463 |
| Medical Sciences | 0.020 | 0.44 | | 0.659 |
| Microbiology | 0.020 | 0.50 | | 0.616 |
| Neuroscience | -0.034 | -0.99 | | 0.324 |
| Pharmacology | 0.057 | 0.65 | | 0.514 |
| Physiology | -0.003 | -0.05 | | 0.96 |
| Plant Biology | -0.002 | -0.05 | | 0.958 |
| Population Biology | 0.069 | 2.42 | | 0.016 |
| Psychology | 0.239 | 4.50 | | <0.001 |
| Physical Science | 0.036 | 0.59 | | 0.557 |
| Social Science | -0.136 | -4.11 | | <0.001 |
| Intercept | 2.012 | 58.06 | | <0.001 |
| N=270, R^2^=0.142. Ordinary least squares regression with robust standard errors. For binary variables, 0=No 1=Yes. Variables for topics which have no papers in the 2009 top 10% are dropped. | | | | |
| Dependent variable: Log10(Citations2009+1) | |  |  | |

*4. Analysis of page-views as of October 2006*

Considering all 2695 papers, we find no significant differences between page-views received by Direct submissions, Communicated papers and Contributed papers (Table S10). When focusing our attention on the 10% most viewed papers from each Track, however, we see that both the top Contributed and Communicated papers receive significantly more page-views than the top Direct submissions (Table S11).

| **Table S10.** Page-views as of October 2006. | | | | |
| --- | --- | --- | --- | --- |
|  | Coefficient | Test statistic | | P-value |
| Communicated | 0.007 | 0.69 | | 0.492 |
| Contributed | 0.015 | 1.42 | | 0.157 |
| Open Access | 0.139 | 10.64 | | <0.001 |
| Special Feature | 0.107 | 3.25 | | 0.001 |
| Age (Days relative to newest article) | 0.0003 | 7.51 | | <0.001 |
| Agricultural Sciences | 0.151 | 2.33 | | 0.02 |
| Anthropology | 0.054 | 1.21 | | 0.225 |
| Applied Biological Sciences | 0.144 | 3.33 | | 0.001 |
| Biophysics and Computational Biology | -0.032 | -1.88 | | 0.06 |
| Cell Biology | 0.107 | 5.92 | | <0.001 |
| Developmental Biology | 0.047 | 1.94 | | 0.053 |
| Ecology | 0.015 | 0.57 | | 0.568 |
| Environmental Sciences | 0.059 | 1.65 | | 0.1 |
| Evolution | 0.009 | 0.47 | | 0.639 |
| Genetics | 0.095 | 4.17 | | <0.001 |
| Immunology | 0.049 | 2.64 | | 0.008 |
| Medical Sciences | 0.111 | 6.51 | | <0.001 |
| Microbiology | 0.020 | 1.00 | | 0.318 |
| Neuroscience | 0.041 | 2.44 | | 0.015 |
| Pharmacology | 0.033 | 1.02 | | 0.307 |
| Physiology | -0.009 | -0.34 | | 0.734 |
| Plant Biology | 0.086 | 3.67 | | <0.001 |
| Population Biology | -0.022 | -0.34 | | 0.732 |
| Psychology | -0.087 | -1.34 | | 0.182 |
| Physical Science | -0.079 | -3.41 | | 0.001 |
| Social Science | -0.057 | -1.40 | | 0.162 |
| Intercept | 3.292 | 225.87 | | <0.001 |
| N=2695, R^2^=0.124. Ordinary least squares regression with robust standard errors. For binary variables, 0=No 1=Yes. | | | | |
| Dependent variable: Log10(Pageviews2006) | |  |  | |

| **Table S11.** Regression on 10% most viewed papers from each track, 2006 page-views | | | | |
| --- | --- | --- | --- | --- |
|  | Coefficient | Test statistic | | P-value |
| Communicated | 0.065 | 3.52 | | 0.001 |
| Contributed | 0.098 | 5.17 | | <0.001 |
| Open Access | 0.030 | 1.47 | | 0.144 |
| Special Feature | -0.068 | -1.69 | | 0.092 |
| Age (Days relative to newest article) | 0.0001 | 1.71 | | 0.088 |
| Agricultural Sciences | -0.042 | -1.82 | | 0.071 |
| Anthropology | -0.064 | -2.90 | | 0.004 |
| Applied Biological Sciences | 0.061 | 1.26 | | 0.209 |
| Biophysics and Computational Biology | -0.015 | -0.50 | | 0.618 |
| Cell Biology | 0.003 | 0.13 | | 0.893 |
| Developmental Biology | 0.060 | 1.42 | | 0.158 |
| Ecology | 0.004 | 0.11 | | 0.913 |
| Environmental Sciences | - |  | |  |
| Evolution | -0.011 | -0.38 | | 0.706 |
| Genetics | 0.087 | 2.34 | | 0.02 |
| Immunology | 0.017 | 0.66 | | 0.513 |
| Medical Sciences | 0.033 | 1.30 | | 0.196 |
| Microbiology | 0.071 | 1.97 | | 0.05 |
| Neuroscience | 0.063 | 1.68 | | 0.094 |
| Pharmacology | -0.063 | -3.56 | | <0.001 |
| Physiology | 0.112 | 5.69 | | <0.001 |
| Plant Biology | 0.007 | 0.23 | | 0.818 |
| Population Biology | -0.033 | -1.66 | | 0.098 |
| Psychology | 0.488 | 22.99 | | <0.001 |
| Physical Science | 0.088 | 2.80 | | 0.006 |
| Social Science | - |  | |  |
| Intercept | 3.710 | 156.19 | | <0.001 |
| N=270, R^2^=0.257. Ordinary least squares regression with robust standard errors. For binary variables, 0=No 1=Yes. Variables for topics which have no papers in the top 10% most viewed are dropped. | | | | |
| Dependent variable: Log10(Pageviews2006) | |  |  | |
